# Supplementary figures and images for: Application of elastic net regression for modeling COVID-19 sociodemographic risk factors
Source: PLoS One. 2024 Jan 26;19(1):e0297065. doi: 10.1371/journal.pone.0297065 (PMC10817220; doi:10.1371/journal.pone.0297065)

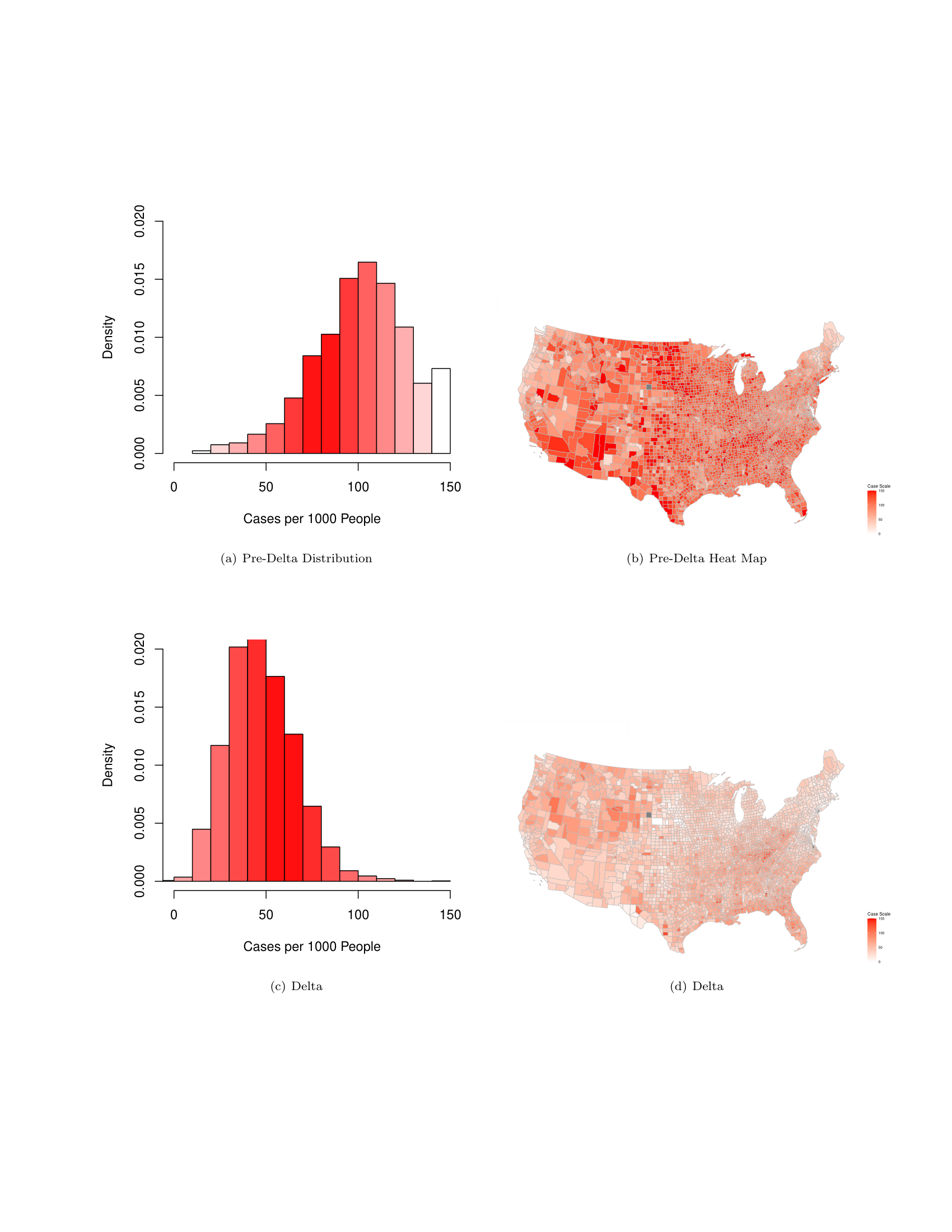

Supplement: S1 Fig — A: Pre-Delta Distribution. B: Pre-Delta Heat Map. C: Delta Distribution. D: Delta Heat Map. (TIFF) [file pone.0297065.s001.tiff]

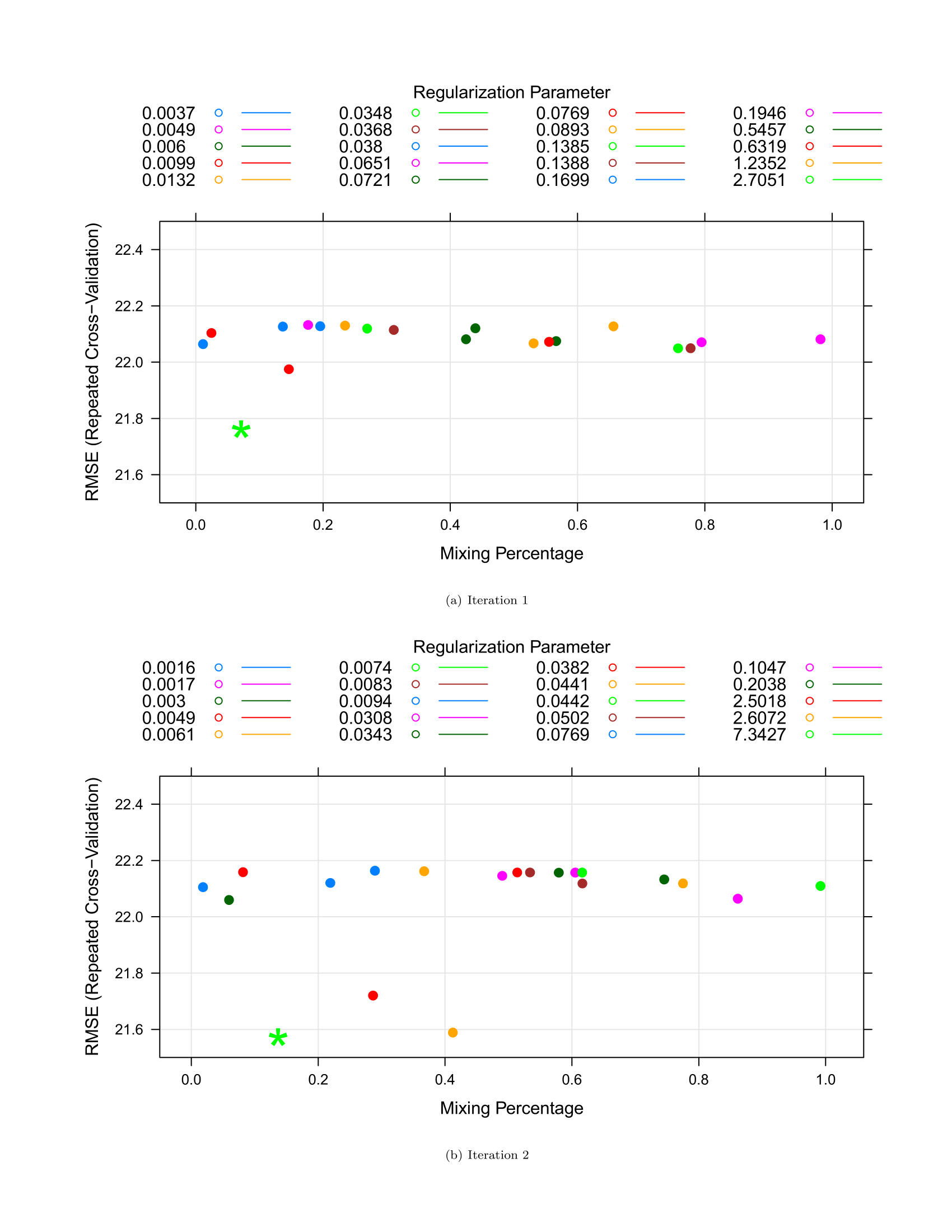

Supplement: S2 Fig — A: Iteration 1. B: Iteration 2. (TIFF) [file pone.0297065.s002.tiff]

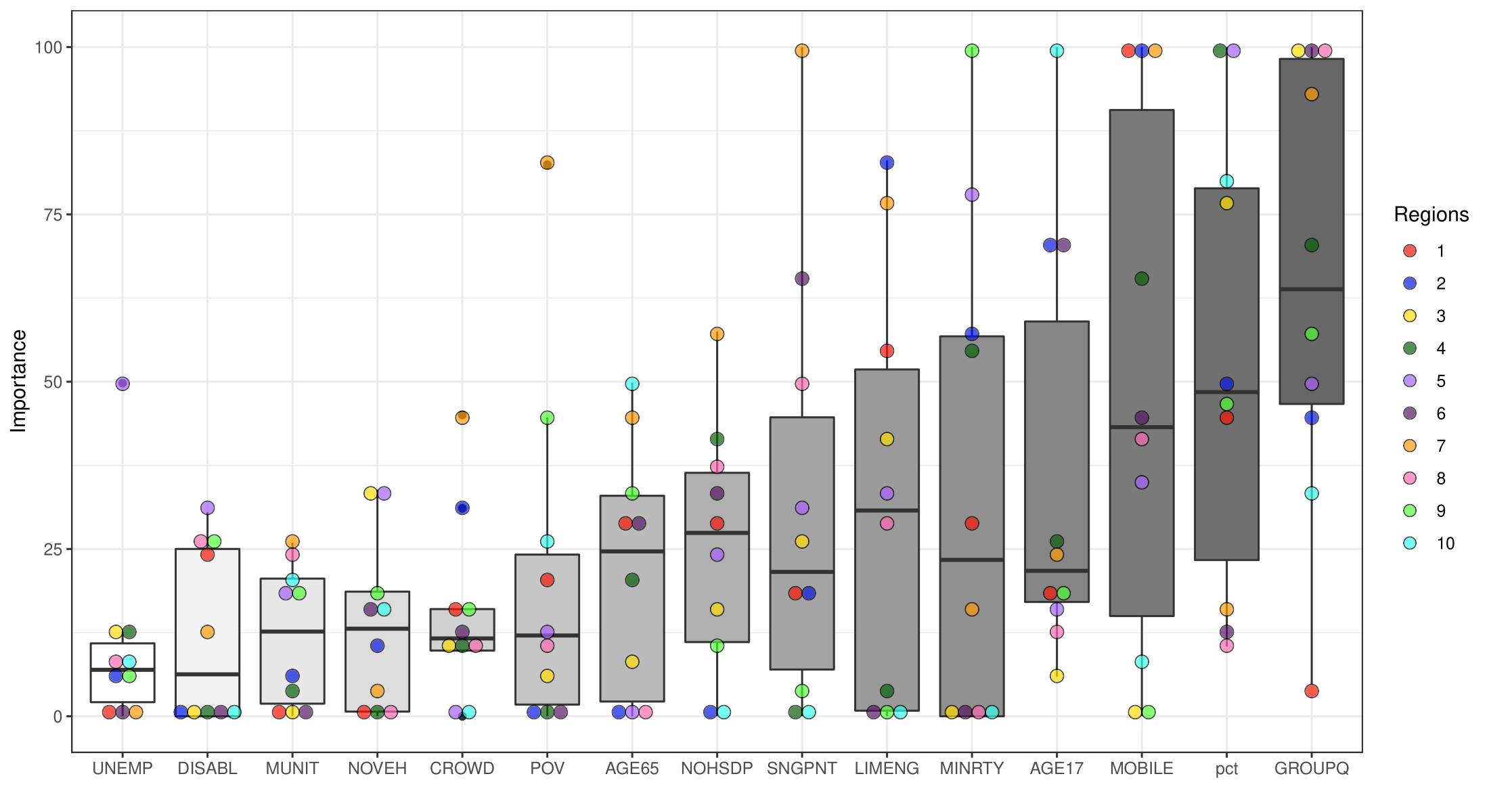

Supplement: S3 Fig — (TIFF) [file pone.0297065.s003.tiff]

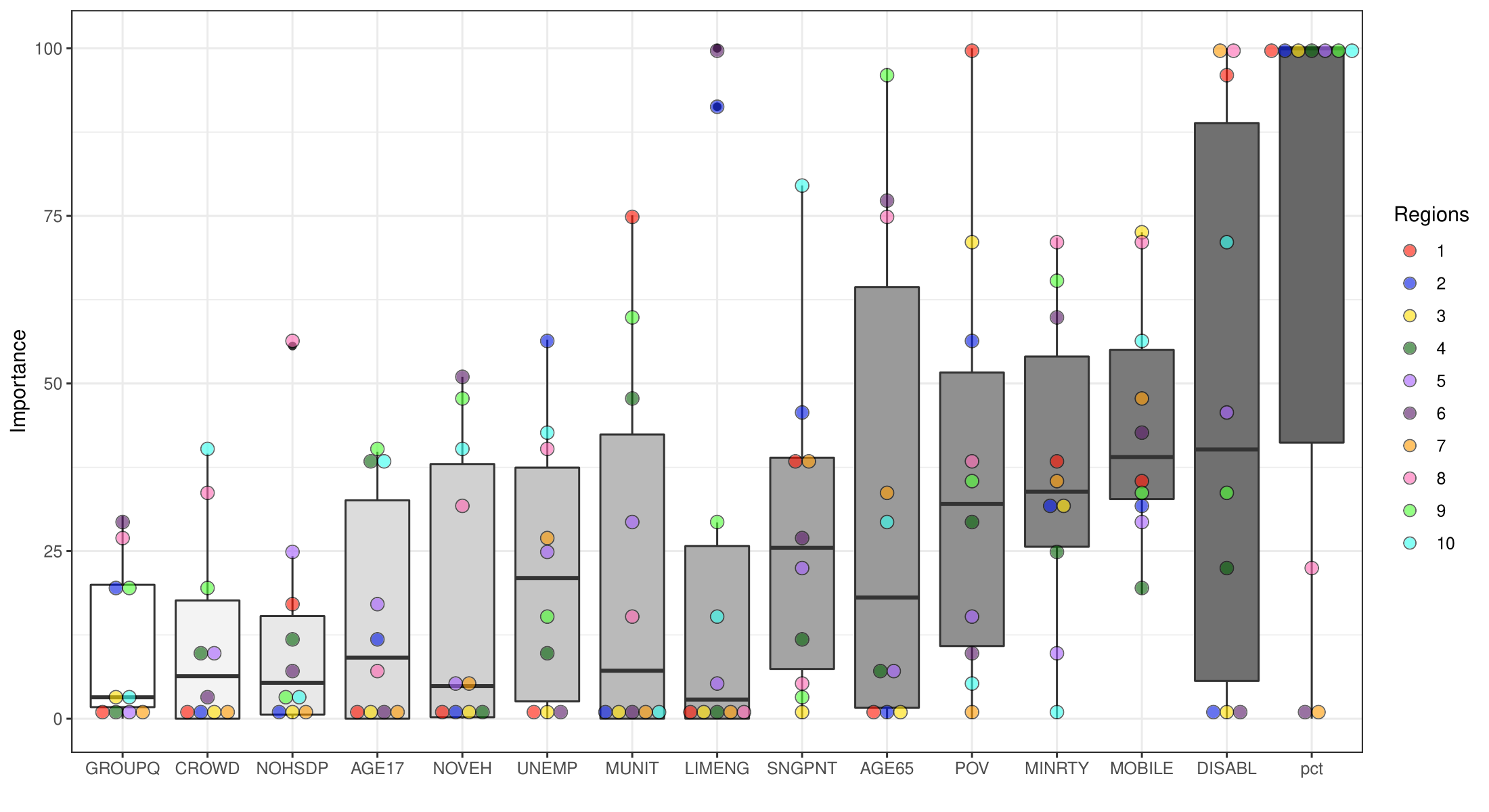

Supplement: S4 Fig — (TIFF) [file pone.0297065.s004.tiff]
