# Supplementary material for: Application of elastic net regression for modeling COVID-19 sociodemographic risk factors
Source: PLoS One. 2024 Jan 26;19(1):e0297065. doi: 10.1371/journal.pone.0297065 (PMC10817220; doi:10.1371/journal.pone.0297065)
Supplement: S1 Appendix — (PDF) [file pone.0297065.s005.pdf]

S1 Appendix. Elastic Net Regression Overview. Elastic net regression is a regularization technique developed by Hui Zou and Trevor Hastie to overcome the limitations of l_1 (lasso) and l_2 (ridge) regularization [19]. l_1 regularization is a penalty to the OLS estimators that results in feature selection, selecting against unimportant variables, whereas l_2 regularization is a penalty to the OLS (Ordinary Least Squares) estimators that shrinks correlated predictors towards each other to overcome multicollinearity [19]. What elastic net achieves is a balance between these two penalties, resulting in a process by which models can be created that deal with multicollinearity among explanatory variables while simultaneously selecting the important features out of a large set of potential predictors. Zou and Hastie (paired with simplified derivations from [43]) describe the set up for the model as follows:

Suppose we have a vector $\mathbf{Y} = (y_1, ..., y_n)$, which is our observed data, and $\mathbf{X} = (x_1| \; ... \; |x_k)$ be our model matrix. Also suppose we have $\mathbf{x}_j = (x_{1j}, ..., x_{nj})^T$, $j = 1, ..., p$ are the predictor variables. We can construct a regression model such that $\mathbf{Y} = \beta_0 + x^T\beta + \epsilon$, where β_0 is the intercept of the regression equation, β are the coefficients for the p explanatory variables, and ϵ is our residual vector. For the elastic net penalty, we assume that the response is centered and the predictors are standardized. This means that the sum of our observed data should equal 0, the sum of our explanatory variables should equal 0, and the mean squared error of our explanatory variables should equal 1. This can be represented as:

$$\sum_{i=1}^{n} y'_i = 0, \qquad \sum_{i=1}^{n} x'_{ij} = 0, \qquad \frac{1}{n}\sum_{i=1}^{n}(x'_{ij})^2 = 1, \qquad j = 1, ..., p$$

Here, y'_i is the centered observed response for the ith county, and x'_{ij} is the standardized measurement for the jth explanatory variable in the ith county. Once this pre-processing has been accomplished, we define the model. The proposition for this model is similar to that of a standard regression model. In standard OLS, we wish to minimize the sum of squared residuals in order to fit a linear model to the data. This is similar to that approach, but with the added l_1 and l_2 penalties. As such, we wish to find:

$$\hat{\beta} = \underset{(\beta_0,\beta)}{\text{argmin}} \; R_\lambda(\beta_0, \beta) = \underset{(\beta_0,\beta)}{\text{argmin}} \left[\frac{1}{2n} \sum_{i=1}^{n} (y_i - \beta_0 - x_i^T\beta)^2 + \lambda P_\alpha(\beta) \right]$$

$$P_\alpha(\beta) = (1-\alpha)\frac{1}{2}\|\beta\|_{l_2}^2 + \alpha\|\beta\|_{l_1}$$

$$= \sum_{i=1}^{p} \left[\frac{1}{2}(1-\alpha)\beta_j^2 + \alpha|\beta_j| \right]$$

α and λ are the hyperparameters for this model, and help to determine the mixture and weight of penalization, respectively. Intuitively, we can see why elastic net is a generalization of lasso and ridge. Setting $\alpha = 1$ results in the elastic net penalty only contributing a penalty equivalent to the magnitude of the β coefficients (also known as the l_1 norm), thus resulting in strictly l_1 regularization. Conversely, setting $\alpha = 0$ results in the elastic net penalty only contributing a penalty equivalent to the squared Euclidean distance of the β coefficients (also known as the l_2 norm), thus resulting in strictly l_2 regularization. These penalties together are scaled by λ; the higher the value for λ, the higher the penalty accrued to the regression.
