# Supplementary material for: Application of elastic net regression for modeling COVID-19 sociodemographic risk factors
Source: PLoS One. 2024 Jan 26;19(1):e0297065. doi: 10.1371/journal.pone.0297065 (PMC10817220; doi:10.1371/journal.pone.0297065)
Supplement: S1 Table — (PDF) [file pone.0297065.s006.pdf]

Table S1. Coefficients and metrics for the 10 HHS regions for the pre-Delta COVID-19 time period, recorded from March 22, 2020 to June 15, 2021

Coefficients										
Region	1	2	3	4	5	6	7	8	9	10
(Intercept)	65.14	87.20	85.06	109.20	106.13	107.57	106.38	113.58	88.21	71.52
In Poverty	−2.46	–	−0.77	–	−1.43	–	−5.51	−1.80	10.72	3.16
Unemployed	−0.14	0.40	−1.28	−1.19	−5.22	–	–	1.27	1.54	−1.07
No HS Diploma	3.70	–	−1.66	3.82	−2.63	2.17	3.84	6.36	2.35	–
Over 65	−3.63	–	−0.97	−1.89	–	−1.91	2.92	–	8.18	−6.14
Under 17	2.30	5.55	0.65	2.50	1.77	4.55	1.67	2.24	4.27	12.32
Disability	−2.95	–	–	–	3.21	–	−0.84	−4.60	−6.11	–
Single Parent	2.17	−1.42	2.78	–	3.33	4.19	6.68	8.36	0.84	–
Minority	3.71	4.59	0	5.07	8.18	–	−1.13	–	24.01	–
Limited English	6.87	6.20	4.60	0.31	3.53	–	5.19	−4.76	–	–
Multi-Unit Home	–	0.56	−0.14	0.36	2.02	–	1.73	3.96	4.40	2.59
Mobile Housing	−12.45	−7.95	0	−6.16	−3.67	−2.85	−6.61	−7.19	–	−1.03
Crowded Housing	−2.00	2.47	1.05	1.08	–	0.76	3.01	1.80	−3.81	–
No Vehicle	–	−0.89	3.58	–	−3.55	1.06	−0.19	–	−4.65	−1.85
Group Quarters	−0.59	−3.61	11.00	6.66	5.29	6.43	6.21	17.06	13.59	4.11
Voting Percentage	−5.68	−3.99	−8.33	−9.38	−10.50	−0.87	−1.07	−1.77	−11.21	−9.85

Metrics										
α	0.10	0.19	0.5	0.91	0.36	0.54	0.73	0.81	0.27	0.42
λ	4.31	2.61	0.46	0.20	0.09	2.10	0.37	0.99	0.81	3.58
ENR Train R^2	0.78	0.78	0.44	0.20	0.41	0.28	0.35	0.40	0.71	0.66
ENR Test R^2	0.78	0.78	0.42	0.15	0.37	0.24	0.35	0.37	0.64	0.64
MR Train R^2	0.82	0.84	0.50	0.22	0.39	0.30	0.38	0.48	0.73	0.75
MR Test R^2	0.76	0.63	0.34	0.11	0.32	0.20	0.30	0.25	0.46	0.60
ENR Test RMSE	15.48	11.52	12.88	20.00	15.68	23.95	21.05	28.11	26.79	19.27
MR Test RMSE	17.49	16.66	13.12	20.09	15.67	33.60	21.10	29.25	28.43	19.52
