# Supplementary material for: Application of elastic net regression for modeling COVID-19 sociodemographic risk factors
Source: PLoS One. 2024 Jan 26;19(1):e0297065. doi: 10.1371/journal.pone.0297065 (PMC10817220; doi:10.1371/journal.pone.0297065)
Supplement: S2 Table — (PDF) [file pone.0297065.s007.pdf]

Table S2. Coefficients and metrics for the 10 HHS regions for the Delta COVID-19 time period, recorded from June 15, 2021 to November 1, 2021

Coefficients										
Region	1	2	3	4	5	6	7	8	9	10
(Intercept)	23.50	24.60	49.69	61.08	40.32	49.07	36.03	39.92	40.47	46.76
In Poverty	1.38	1.25	5.15	2.82	1.17	0.31	–	−2.51	2.69	−0.30
Unemployed	–	1.26	–	1.01	−2.09	–	1.01	2.72	−1.23	2.53
No HS Diploma	0.23	–	–	−1.19	−1.96	0.24	–	−3.69	0.19	−0.20
Over 65	–	–	–	0.76	−0.52	−2.49	−1.26	−4.95	-7.41	−1.68
Under 17	–	0.26	–	3.73	1.44	–	–	−0.44	3.04	2.21
Disability	1.31	–	–	2.20	3.77	–	3.76	6.64	2.57	4.16
Single Parent	0.52	1.00	–	1.12	1.87	0.90	1.48	−0.40	0.23	4.70
Minority	−0.53	−0.69	−2.30	−2.34	0.85	−1.91	−1.34	−4.76	−4.99	–
Limited English	–	−2.03	–	–	−0.44	−3.23	–	0.02	−2.23	−0.90
Multi-Unit Home	−1.03	–	–	4.58	2.36	–	–	0.95	−4.62	–
Mobile Housing	0.49	−0.72	5.21	1.81	2.35	1.37	1.83	4.74	−2.61	3.37
Crowded Housing	–	–	–	0.88	−0.74	0.12	–	2.20	−1.56	−2.33
No Vehicle	–	–	–	−0.09	0.43	1.65	0.17	2.17	3.61	2.35
Group Quarters	–	−0.45	−0.29	0.19	0.133	−0.98	–	−1.73	−1.46	0.14
Voting Percentage	−1.37	−2.23	−7.18	−9.78	−8.09	–	–	−1.45	−7.64	−5.91

Metrics										
α	0.30	0.84	0.71	0.66	0.67	0.90	0.95	0.12	0.14	0.20
λ	1.94	0.19	1.76	0.14	0.07	0.78	0.60	0.98	0.98	1.18
ENR Train R^2	0.47	0.62	0.72	0.46	0.55	0.16	0.24	0.28	0.73	0.60
ENR Test R^2	0.46	0.55	0.72	0.41	0.51	0.16	0.27	0.25	0.69	0.59
MR Train R^2	0.61	0.72	0.84	0.47	0.57	0.21	0.31	0.30	0.76	0.70
MR Test R^2	0.15	0.29	0.57	0.37	0.45	0.07	0.22	0.18	0.62	0.46
ENR Test RMSE	7.04	4.49	12.33	13.09	9.15	15.21	9.96	15.05	9.30	14.09
MR Test RMSE	12.05	4.77	14.83	13.16	9.18	14.92	10.36	16.04	9.79	14.86
