# Supplementary material for: Application of elastic net regression for modeling COVID-19 sociodemographic risk factors
Source: PLoS One. 2024 Jan 26;19(1):e0297065. doi: 10.1371/journal.pone.0297065 (PMC10817220; doi:10.1371/journal.pone.0297065)
Supplement: Attachment — Submitted filename: Review Plos One 2023.docx [file pone.0297065.s008.docx]

Thank you for the opportunity to review the manuscript entitled “Application of Elastic Net Regression for Modeling COVID-19 Sociodemographic Risk Factors”. The article is of interest for the readership of PLOS ONE as it focuses on factors of influence for case rate of COVID-19.

First, in my opinion, the authors failed to explain the reason for which constructing 10 models (one per region) is a preferred approach to 1 general model - for each time frame. While it is clearly explained why the authors split the analysis into the 2 time periods, the option of looking separately at each region is not argued in a proper manner.

Second, the variations in terms of health care infrastructure as a proxy for preventive measures could be also a relevant variable of influence. The authors should consider discussing it (at least in the limitation section).

I hope that my comments are useful for authors, as they further develop the study.
